# Predictive limitations of spatial interaction models: a non-Gaussian analysis

**DOI:** 10.1038/s41598-020-74601-z

**Published:** 2020-10-15

**Authors:** B. Hilton, A. P. Sood, T. S. Evans

**Affiliations:** Centre for Complexity Science and Theoretical Physics Group, Physics Department, Imperial College London, London, SW7 2AZ UK

**Keywords:** Statistical physics, thermodynamics and nonlinear dynamics, Complex networks, Scientific data

## Abstract

We present a method to compare spatial interaction models against data based on well known statistical measures that are appropriate for such models and data. We illustrate our approach using a widely used example: commuting data, specifically from the US Census 2000. We find that the radiation model performs significantly worse than an appropriately chosen simple gravity model. Various conclusions are made regarding the development and use of spatial interaction models, including: that spatial interaction models fit badly to data in an absolute sense, that therefore the risk of over-fitting is small and adding additional fitted parameters improves the predictive power of models, and that appropriate choices of input data can improve model fit.

## Introduction

The ability to predict the number of vehicles, the amount of goods, or the spread of disease between two locations, using only limited data about each location, is important in a variety of academic disciplines. Problems of this nature can be studied using ‘spatial interaction models’. Given some measures of the importance of each site *i*, and the distance $d_{ij}$ between two sites *i* and *j*, these models predict the flow from site *i* to site *j*, denoted $F_{ij}$. The distance $d_{ij}$ need not be a geographical distance; it could reflect the cost of travel or other socio-economic measures of separation. These models only predict flows between distinct sites, and so $i \ne j$.

The nature of spatial interaction models and the associated data means that residual errors cannot always be assumed to be Gaussian, though this is often assumed in the literature. Our primary goal is to improve upon the statistical analysis commonly carried out in the literature and apply this improved analysis to determine the relative effectiveness of key examples from two popular families of models: gravity models and radiation models. Additionally, our methods are used to identify which features of these models give the greatest improvement in results.

We will start by reviewing the data used in our work. In “Models” section, we will look at the various spatial interaction models we consider. The statistical methods used are described in “Statistical methods” section with more details on alternatives used in the literature given in Appendix D of the Supplementary Information. Our results are then shown in “Results” section. We will conclude with a discussion of our work. A summary of the notation used in this paper is provided in Appendix A of the Supplementary Information.

## Data

It is inherent to the nature of statistical analysis that models must be compared against data. In this paper we wish to focus on the features of spatial models and on the features of different analysis methods used to study spatial data and models. To do this we sought a dataset which acts as a standard to be used when comparing different models and different analysis techniques. It is essential then that such a standard is an open data set and it would be useful if the standard dataset was already well known and well studied to give authors many sources of independent information on the standard dataset. We have chosen to work with the US Census 2000: the county-to-county worker flow data from the US Census 2000^1^. It is both an open source dataset and widely used.

In particular, the US Census 2000 datset was used in Simini et al.^2^ when developing the Radiation model. This ensures that any differences between our results and those of Simini et al.^2^ arise due to changes in the analysis rather than simply the choice of data. Using this data, the radiation model was compared favourably against the gravity model^2^.

As a further check and to verify that our conclusions are a result of the models and the US commuter flow system rather than merely a feature of the specific data set, we also used the parallel data set from the American Community Survey^3^ 2009–2013. We obtained the populations of the counties at the census dates of 2000^4^ and 2010^5^. Though we often use the language of commuting to describe our approach, our methods are data set agnostic, and therefore our results have wider applicability.

**Figure 1:**
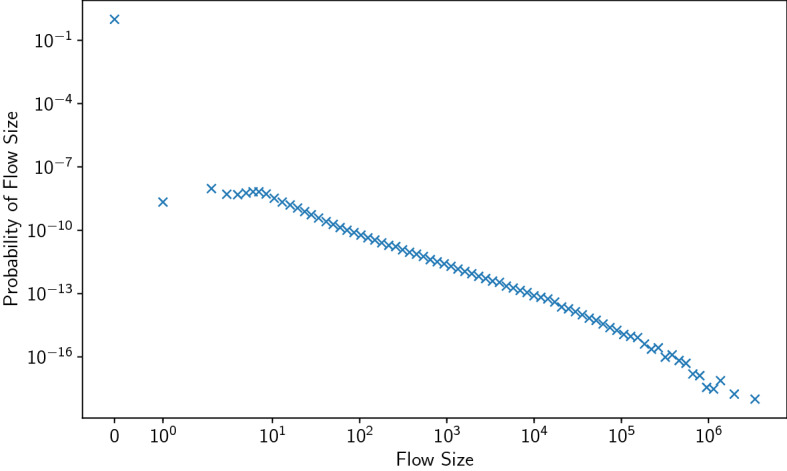
The distribution of commuter flow sizes in the US Census 2000 data^1^.

In the US Census 2000, there are 3109 counties or their equivalents within the 48 contiguous United States. These form the sites used by our spatial models. The US Census 2000 asked, for each person listed: “at what location did this person work *last week*?” Respondents were further instructed “if this person worked at more than one location, [to] print where he or she worked most last week.” This means that our figures for commuting will include data from those who occasionally work at other locations for a few days and these are likely to inflate the number of long distance trips recorded relative to data representing where a person worked for most of a year. Information on the distribution of flows is shown in Fig. 1 and Appendix E of the Supplementary Information.

From this data we define three values associated with each site *i*, which are generic to many spatial interaction contexts: the site population $P_i$, the flow into a site $I_i$, and the flow out $O_i$. While these three values are likely to be correlated at each site for our commuting data, there are large individual differences as sites may have developed specialised functions. For instance in the US Census 2002 data^1^, many people work in San Francisco county who commute in from other counties (265,291 people), but fewer live in San Francisco county and commute elsewhere (130,036 people).

We use this data on the population and the number of commuters arriving and leaving a site to determine model parameters associated with site importance. We use $w_i$ (site weight) as a generic site importance model parameter but, depending on the model, we can use up to three more specific site parameters to characterise a site: a repulsiveness parameter $t_i$ controlling the total flow out of a site, an attractiveness parameter $n_i$ that controls the flow into a site, and in some cases an ‘aspiration’ parameter $m_i$ that controls how far a commuter will travel.

The distances needed for the models were great-circle distances between the geographical centres of each pair of US counties. These data were obtained from the National Bureau of Economic Research^6^.

## Models

### Gravity models

One of the most widely used spatial interaction models is a class of models known as ‘gravity models’, which have been used in a variety of socio-economic contexts since the 19th Century but have seen much development since the 1950s (see elsewhere^7,8^ for general reviews).

The simplest gravity model is given by

1 $$\begin{aligned} {\widehat{F}}_{ij} = w_i w_j f(d_{ij}) \, , \end{aligned}$$

where ${\widehat{F}}_{ij}$ is the model’s estimate of the flow $F_{ij}$ from *i* to *j*. The $w_i$ and $w_j$ parameters are the weights of sites *i* and *j* respectively, some measure of the importance of sites. The function $f(d_{ij})$ is some monotonically decreasing function of (generalised) distance: the ‘deterrence function’. This function is often chosen without theoretical motivation and typically includes additional parameters; these must be determined using previously known data. Such flexibility in the form of the deterrence function can be regarded as a key limitation of the gravity model^2^. However, in practice simple forms are often found to be effective. Common deterrence functions include exponentials^9^ ($f(x) = e^{-\beta x}$ for some $\beta > 0$) and power laws^10,11^ ($f(x) = x^{-\beta }$ for some $\beta > 0$). The deterrence functions invariably include a global parameter, $\beta$ in our examples, that is the same for all pairs of sites. This might be set from data, for instance $\beta ^{-1}$ represents a typical length scale for the exponential form. However, such global model parameters are often determined by varying their values until the model has the best possible fit to the data.

In order to accurately test the extent of the difference in predictive power between models, they must share any feature that is not being explicitly compared. All the models considered here are ‘production constrained’ models in which the output of each site is fixed by a model parameter for that site. So rather than the simplest gravity model of Eq. (1), we will use a production constrained gravity model^7,12,13^ of the form

2 $$\begin{aligned} {\widehat{F}}_{ij} = \frac{ t_i n_j d_{ij}^{-\beta }}{\sum _k n_k d_{ik}^{-\beta }} \, . \quad (i \ne j) \end{aligned}$$

This obeys $\sum _j {\widehat{F}}_{ij} = t_i$, the production constraint making the site model parameter $t_i$ equal to the total flow leaving site *i*. The $n_j$ parameter is some measure of the ‘attractiveness’ of site *j* that controls the flow into each site, though this is not necessarily equal to the flow into site *j*. Even if $t_i=n_i$ (as is often assumed), it is worth noting that this model already describes an asymmetric flow with ${\widehat{F}}_{ij}\ne {\widehat{F}}_{ji}$ in general. Thus, unlike the simple gravity model, this production constrained gravity model can produce flow asymmetries akin to those that are present in real data, as illustrated by the example of San Francisco county considered in “Data” section.

For our work with the gravity model Eq. (2), we will set the output site parameter equal to the number of commuters leaving a site, $t_i=O_i$, while the site attractiveness parameter will be set equal to the number of commuters arriving $n_i=I_i$. We will choose the single global model parameter $\beta$ in Eq. (2) to be the value that gives the best fit to our data as explained below. For comparison, the gravity model against which the radiation model is compared in Simini et al.^2^ also used a power law deterrence function, but had no constraints on inputs or outputs, and used nine fitted parameters (see Appendix C of the Supplementary Information).

Other forms for the deterrence function in our gravity model were also investigated, but the power law in Eq. (2) proved the fairest comparison^14,15^.

### The radiation model

The radiation model was derived in the context of commuter flows, using the underlying assumption that a worker seeking employment will accept the most proximate job offer that meets their requirements. The most general form of the radiation model used by Simini et al.^2^ is

3 $$\begin{aligned} {\widehat{F}}_{ij} = t_i \frac{m_i n_j}{(m_i + s_{ij})(m_i + n_j + s_{ij})} \, . \end{aligned}$$

The model parameter $t_i$ controls the total flow leaving each site *i* and we have that $\sum _j {\widehat{F}}_{ij} \approx t_i$ making this radiation model a production constrained model. We will return to this approximation below. The $n_i$ model parameter is the number of opportunities drawing commuters into site *i*, the site attractiveness parameter in this model. The $s_{ij}$ is given by the sum of all opportunities of sites closer to *i* than *j*, the intervening opportunities measure^16^

4 $$\begin{aligned} s_{ij} = \sum _{k | k \ne i} n_k \theta (d_{ij} - d_{ik}) \, . \end{aligned}$$

Here $\theta (x)$ is one for $x>0$ and zero otherwise so the sum does not include $n_i$ or $n_j$. The last model parameter $m_i$ is a measure of the aspiration of commuters leaving site *i*. That is, the larger the value of $m_i$, the greater the aspirations of the commuters leaving site *i*, and the further they must travel to achieve their aspirations. Thus, $m_i$ does not alter the total flow leaving site *i*, but $m_i$ controls the distribution of the flow leaving site *i*.

We noted above that the flow leaving each site *i* is not exactly equal to the $t_i$ model parameter. This is easily corrected^17^ and by writing Eq. (3) using a partial fraction decomposition, we arrive at a normalised form of the radiation model

5 $$\begin{aligned} {\widehat{F}}_{ij} = \left( \frac{N_c}{N_c-m_i}\right) t_i \frac{m_i n_j}{(m_i + s_{ij})(m_i + n_j + s_{ij})} \, . \end{aligned}$$

Here $N_c= \sum _i n_i$ is the total number of opportunities in the system. With this normalisation, the production constraint is perfectly enforced in the normalised radiation model, $\sum _j {\widehat{F}}_{ij} = t_i$. If $N_c \gg n_i,m_i$ then this normalised radiation model form is almost the same as Eq. (3) showing this correction (the factor in brackets) is often small.

One of the important features of the radiation model is that the form is fixed; there is no equivalent here to the choice of deterrence function seen in gravity models. This means there are no explicit global model parameters in the radiation model, such as the $\beta$ in Eq. (2). The lack of such global model parameters (as opposed to those parameters linked to site properties) leads to the description of the radiation model as having a “parameter-free nature”^2^.

However, to use the radiation model, or indeed any spatial interaction models, we must first relate the site model parameters to values in our data. Mapping these site model parameters to data values can be done in many ways and this leads to a family of radiation models. The versions of the radiation model analysed here are summarised in Table 1, with more details given in Appendix B of the Supplementary Information. In particular, the original radiation model^2^ used the total population $P_i$ of site *i* to set the three site model parameters with $m_i=n_i=P_i$ and $t_i = \alpha P_i$: model F in Table 1 (see also (B.8) in Supplementary Information). Note that $\alpha$ is a single fitted global model parameter, exemplifying how such parameters can be introduced to spatial interaction models through the mapping of data to model parameters. In such a case, even the radiation model is no longer parameter free in the sense defined above. In our examples only our radiation models A to E are parameter free, the remaining radiation models and our gravity model both have one fitted global model parameter.

The radiation model has been widely used in the literature as the basis for a variety of other models^18–20^. We will focus on the family of models described above that include only minor changes to the original radiation model in order to draw conclusions about the effects of each of these changes.

**Table 1:** A summary of the different versions of the radiation model.

Name	${{\text{m}}_{\text{i}}}$	${{\text{n}}_{\text{i}}}$	${{\text{t}}_{\text{i}}}$	Normalised?	Eq.
A. Total population	$P_i$	$P_i$	$P_i$	$\times$	(B.3)
B. Departing commuters	$O_i$	$O_i$	$O_i$	$\times$	(B.4)
C. Departing commuters, normalised	$O_i$	$O_i$	$O_i$	$\checkmark$	(B.5)
D. Arriving & Departing, Naïve split	$O_i$	$I_i$	$O_i$	$\times$	(B.6)
E. Arriving & Departing, Revised split	$I_i$	$I_i$	$O_i$	$\checkmark$	(B.7)
F. Total population, fitted factor	$P_i$	$P_i$	$\alpha P_i$	$\times$	(B.8)
G. Departing commuters, fitted factor	$O_i$	$O_i$	$\alpha O_i$	$\times$	(B.9)
H. Arriving & Departing, Revised, Fit factor	$I_i$	$I_i$	$\alpha O_i$	$\checkmark$	(B.10)

The tick in the ‘Normalised?’ column indicates that a model uses a normalisation that enforces the production constraint exactly Eq. (5), while a cross in that column indicates that the original form Eq. (3) is used for that model. In each case we specify which of the site data values, ($P_i$ population, $I_i$ commuters arriving, $O_i$ commuters leaving) is used for the model site parameters (aspirations $m_i$, opportunities $n_i$, out flow $t_i$). See Appendix A of the Supplementary Information for a summary of the notation. The single global model parameter $\alpha$ is found by optimising the fit of the model to the data. The model used by Simini et al.^2^ is equivalent to our model F. The full equations are given in Appendix B of the Supplementary Information as indicated in the final column.

## Statistical methods

There are two statistical challenges when dealing with spatial interaction models and data. Suitable statistical measures must be chosen to evaluate how well the models’ parameters (where present) give the best fit to data, and secondly some metric must be selected to establish which model is ‘best’. However, the choice of this metric is not obvious. For example, one may decide to prioritise accurately predicting which pairs of sites will have zero flow ($F_{ij} = 0$) over gaining accurate estimates of the sizes of large flows. We attempt to sidestep such issues by asking in an unbiased statistical sense how *probable* the models are. In order to achieve this, it is worth first considering some of the techniques found in the existing literature.

### Common techniques for comparing models

A wide range of methods are used to compare spatial data against data^21^ for a study of spatial data and models using many such measures. However, there are problems with the underlying statistical basis for many of the most popular approaches.

The Sørensen-Dice coefficient is often used to compare models against real data^17,18,21–26^ and is sometimes referred to as the ‘common part of commuters’ in this context. This is defined as $\text {DSC} = {\sum _{ij} \min ({\widehat{F}}_{ij}, F_{ij})}/{\sum _{ij} F_{ij}}$ for model values ${\widehat{F}}_{ij}$ and flow data $F_{ij}$ from site *i* to site *j*. One drawback of the Sørensen-Dice coefficient is that small percentage deviations in the predictions of large flows have a significant impact on the Sørensen-Dice coefficient. However the main reason we do not use this measure is that it has no statistical basis; it used elsewhere because of its ‘intuitive explanatory power’ to quote^22^ Gargiulo et al. The Sørensen-Dice coefficient may still be useful but we are looking for a measure whose validity can be assessed apriori with more rigour.

Sometimes a comparison is made using statistics that assume an underlying Gaussian distribution: i.e. where it is assumed that the error distribution $p(F_{ij} | {\widehat{F}}_{ij})$ (the probability that the flow is found to be $F_{ij}$ given a predicted flow ${\widehat{F}}_{ij}$) is Gaussian for any *i*, *j*. A common example of a measure of this type is the coefficient of determination^17,27,28^ $R^2= 1 - {\sum _{ij} (F_{ij} - {\hat{F}}_{ij})^2} /{\sum _{ij} (F_{ij} - {{\bar{F}}})^2}$ but other examples include mean squared errors^29^, and Pearson correlation coefficients^30,31^. However, real data sets give integer valued data, feature no negative flows, and usually have a high proportion of very small flows. A Gaussian model of fluctuations when applied to small pairs of sites with small flows will predict real and sometimes negative flows which are poor approximations (at best) for the actual fluctuations.

The Kolmogorov-Smirnov test is also seen in spatial modelling^20^ and it is defined in terms of $K = \sup |{\widehat{F}}_{ij} - F_{ij}|$. One advantage is that this test does not make assumptions about the distribution of fluctuations in $F_{ij}$ or ${\widehat{F}}_{ij}$. However, the Kolmogorov-Smirnov test does require that the two input functions are independent. Unfortunately, in spatial modelling the parameters of the model are usually estimated by fitting the model to the data so now model values ${\widehat{F}}_{ij}$ and data values $F_{ij}$ are no longer independent. The Kolmogorov-Smirnov test is then invalid and it can produce dangerous results in such circumstances^32^.

Finally, none of these tests measure the effects of fitting parameters: varying a model parameter to fit data can improve the accuracy of the model for that data set, but at the expense of reducing the model’s predictive power on other data sets. Further discussion on these commonly used techniques, as well as an application of these techniques to the models in this paper, can be found in Appendix D of the Supplementary Information.

### Poisson regression

The limitations of these techniques motivate the application of alternative statistical methods^33^. Our starting point is the determination of the error distribution $p(F_{ij}| {\hat{F}}_{ij})$. Were there data on commuting for every day over a few years, we could look at the actual fluctuations in flows and examine the validity of this statistical model. However, without this data, and given that the chosen data sets (see “Data” section) contain discrete count data, the simplest assumption we can make is to assume that the flow $F_{ij}$ between any one pair of sites is Poisson distributed: that for any given pair of sites, we model the probability of finding flow $F_{ij}$ in the data as $p(F_{ij} | {\widehat{F}}_{ij}) = \exp ( -{\widehat{F}}_{ij} ) ({\widehat{F}}_{ij})^{F_{ij}}/(F_{ij}!)$, where we have taken the model estimate ${\widehat{F}}_{ij}$ to be the mean of our distribution. For small flows, the majority of values in our data, this is significantly different from a Gaussian distribution.

In fact, the models used here are built on Poisson processes making this assumption even more appropriate. We can interpret the flows given by gravity models as the flows which maximise a certain entropy function^7,12,13^. This in turn means that we can interpret a Gravity model at a microscopic level as placing discrete trips with a probability specified by the form of the entropy function. Even links with small flows are well described by a Poisson distribution in gravity models. Likewise the Radiation model^2^ is constructed from probabilities that commuters leaving one site will arrive at another, probabilities which are independent of the state of the system. Again the result quoted for flows in the Radiation model is just the mean of a predicted Poisson distribution.

Using these assumptions, we can now ask how probable it is that the data would be observed given the distribution predicted by the model. This is known as ‘Poisson regression’. Using Poisson regression, we calculate the log-likelihood $\ln L$ for model values ${\widehat{F}}_{ij}$, given some flow data $F_{ij}$, where we retain the option to work only with flows above a minimum value $F_\text {min}$, namely

6 $$\begin{aligned} \ln L(F_\text {min}) = \sum _{\begin{array}{c} i,j \\ i \ne j, \end{array} } \left( - {\widehat{F}}_{ij} + F_{ij} \ln ({\widehat{F}}_{ij}) - \ln (F_{ij}!) \right) \theta (F_{ij}-F_\text {min}) \, . \end{aligned}$$

It is important here that the predicted flow in these models is never zero so we we always get a finite result for $\ln L(F_\text {min})$. Log-likelihood functions and maximum likelihood estimations provide a rigorous way to estimate fitted parameters, and to quantitatively compare how well models fit data. While adding more fitted parameters will always improve the fit of the model to the data, this risks over-fitting to the particular data set used, reducing the models’ general predictive power. Thus log-likelihood values cannot tell us whether or not these fitted parameters have truly improved the model, and we need a different measure of model effectiveness.

Ideally, in order to test model effectiveness, a form of cross-validation would be used, wherein the model is fit to some data and then tested against a second data set drawn from the same distribution^34^. However, the difficulty in obtaining multiple real data sets drawn from the same distribution means that some other model selection criterion must be used. One widely-used method is the Bayesian information criterion^35,36^ given by

7 $$\begin{aligned} \text {BIC}(F_\text {min}) = k \ln (n) - 2 \ln (L(F_\text {min})) , \end{aligned}$$

where *k* is the number of fitted parameters, *n* is the number of data points, and *L* is the likelihood. The Bayesian information criterion can be used to compare models against a single common data set. It has a robust statistical basis^37^, introducing a penalty that increases with the number of fitted parameters. This penalty is sometimes considered too harsh^38^.

Finally, it would be useful to have a measure of goodness-of-fit. Log-likelihood (and therefore Bayesian information criterion) values can only be used to compare models. They allow us to say one model matches real data more closely than another, but do not conclude that they resemble real data well in any absolute sense. For this, we need some value against which likelihood values can be compared. One method is to use the saturated likelihood $L_s$: the value that the likelihood would take if the predictions from the model exactly matched the data. The ratio of the actual likelihood to this saturated value must be between zero and one and can be used to define the deviance $D\ge 0$ through $L/L_s = \exp (- D/2 )$. In our case we have that

8 $$\begin{aligned} D(F_\text {min}) = 2 \sum _{\begin{array}{c} i,j \\ i \ne j, \end{array} } \left( ({\widehat{F}}_{ij} - F_{ij}) + F_{ij}\ln ( F_{ij}/{\widehat{F}}_{ij}) \right) \theta (F_{ij}-F_\text {min}) \, . \end{aligned}$$

For all three of these statistics (log-likelihood, BIC and deviance), the lower the magnitude, the better the model fits the data.

## Results

**Figure 2:**
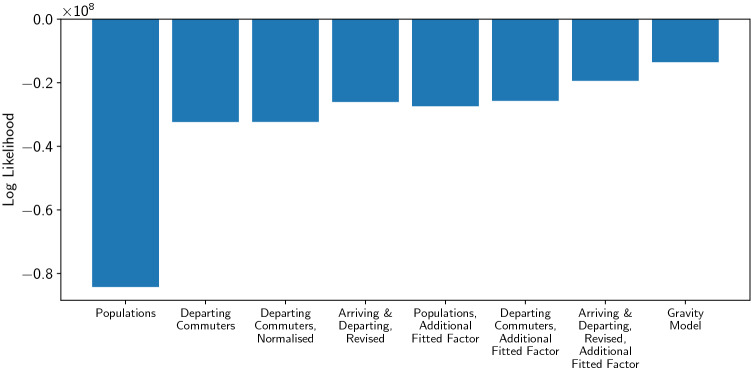
The log-likelihood values Eq. (6) for radiation models A, B, C, E, F, G, H (from left to right) described in Table 1, alongside the production constrained gravity model of Eq. (2). Less negative values represent better models. These data are from the US Census 2000^1^. The uncertainty in the value of any fitted parameter led to a negligible change in these results.

Figure 2 shows the log-likelihoods for the various versions of the radiation model in Table 1, and for the production constrained gravity model of Eq. (2), calculated using the commuting data of the US census 2000^1^. The exact values of the log-likelihoods and associated standard errors are shown in Table 2. Radiation model D (see Table 1) has been omitted from the figures in this section because of its extremely large log-likelihood—it is far worse than any other model. This is unsurprising since this model has assumed that $m_i$ and $n_i$ can be used analogously with $t_i$ and $n_i$ in the gravity model, without any theoretical justification for why this might be the case; an asymmetry is naïvely introduced into the model where the quantities governing site inflow and outflow are disentangled without a derivation matching this to the real world. This result thus acts as a simple check of our approach in dealing with radiation model parameters, rather than the intuitive approach of assuming that any parameters pertaining to the source site *i* are ‘repulsiveness’ measures and parameters pertaining to target site *j* are ‘attractiveness’ measures.

**Table 2:** The log-likelihood values Eq. (6) for the various radiation models of Table 1 and the production constrained gravity model of Eq. (2).

Model	Log-likelihood $\ln L$	Error due to fit
A	$-8.4\times 10^7$	N/A
B	$-3.2 \times 10^7$	N/A
C	$-3.2 \times 10^7$	N/A
D	$-7.0 \times 10^8$	N/A
E	$-2.6 \times 10^7$	N/A
F	$-2.7 \times 10^7$	$1 \times 10^{-4}$
G	$-2.6 \times 10^7$	$3 \times 10^{-4}$
H	$-1.9 \times 10^7$	$3 \times 10^{-4}$
Gravity model	$-1.4 \times 10^7$	$3 \times 10^{-4}$

The standard error in the log-likelihood comes from the uncertainty in the value of any fitted parameters, calculated from the Hessian. Thus we have no estimate of uncertainty for models without a fitted parameter, as indicated by an “N/A” entry.

These log-likelihoods allow for an initial comparison between models. Radiation model A (‘Populations’) is the worst model other than radiation model D. The total flow out of each site in radiation model A is generally significantly larger than real flows, leading to its poor performance. Changing the site model parameters to be equal to the departing commuters data value $O_i$ (radiation model B—‘Departing commuters’) improves the model significantly, as expected. Adding in a normalisation (radiation model C—‘Departing commuters, Normalised’) only results in a slight improvement. This is because of the large number of commuters in the USA; the largest possible value of the normalisation factor is 1.0168 and the mean value is 1.0003. Using a model in which the site model parameters for input and output flow, $n_i$ and $t_i$ respectively, are related to the corresponding data values, $I_i$ and $O_i$ respectively, produces the best results. This is radiation model E—‘Arriving & Departing, Revised’.

Every model with an additional fitted factor works better than its counterpart: F is better than A, G is better than B, and H is better than E. Moreover, even model F (‘Populations, Additional Fitted Factor’), which one might expect would overestimate the flows due to its large site parameter values, arrives at a better log-likelihood than either model B (’Departing Commuters’) or C (‘Departing Commuters-Normalised’). However, model G (‘Departing Commuters, Additional Fitted Factor’) is more successful than model F (‘Populations, Additional Fitted Factor’), indicating that the matching of model site parameters to appropriate site data values still has merit.

The explanation for the particularly strong improvement resulting from fitting lies in the idea, corroborated below, that none of these models fit real data particularly well. Consequently, allowing a parameter to vary until the best possible value is found optimises the models’ effectiveness far more than ensuring model site values are well matched to data when the overall model only approximates reality very roughly. Intriguingly, our gravity model Eq. (2), whose form was chosen so as to be comparable to our radiation models, matches our real data more closely than any of our radiation models.

**Figure 3:**
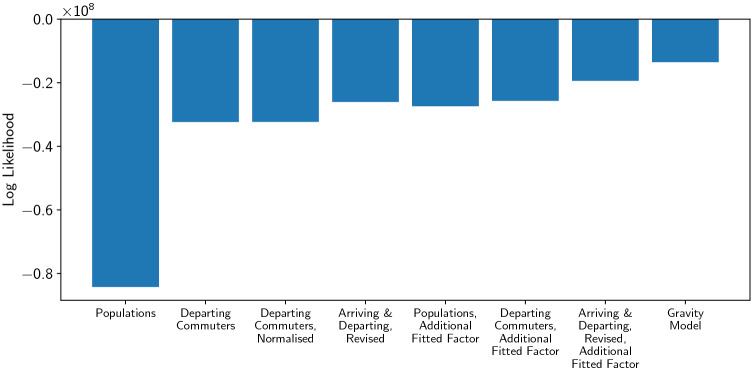
Bayesian information criterion values Eq. (7) for radiation models A, B, C, E, F, G, H (from left to right) described in Table 1, alongside the production constrained gravity model of Eq. (2). Lower values represent better models. These data are from the US Census 2000^1^.

Log-likelihoods alone do not tell the full story. Figure 3 shows the BIC values for the models. Despite the BIC often being regarded as overly harsh with regards to additional parameters^38^, the trend shown is exactly the same as in Fig. 2. This is because the penalty applied by the BIC is $k \ln (n)$, and $\ln (n)$ is only 8.04. This is much smaller than the log-likelihood values of order $10^7$. We can therefore conclude that there is very little risk of over-fitting, and that adding relevant additional fitted parameters significantly improves the models.

**Figure 4:**
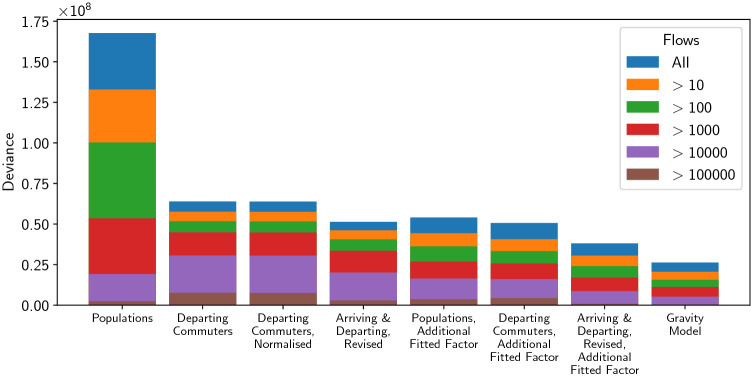
Deviance values Eq. (8) for radiation models A, B, C, E, F, G, H (from left to right) described in Table 1, alongside the production constrained gravity model of Eq. (2), with data sets that are truncated using the minimum values shown in the legend. Lower values represent better models. These data are from the US Census 2000^1^. For each model the top of a coloured bar represents the deviance value for that model when the data is limited to flows above the value indicated in the legend.

Figure 4 shows the deviance values for each model. The blue bars are almost identical in appearance to Fig. 3 because the magnitude of the actual log-likelihood ($\sim 10^7$) far exceeds that of the saturated log-likelihood (~ 40,000). This comparison underscores how poorly these models fit real data in an absolute sense.

Given that most of the data is zero, we might wonder to what extent these trends are an artefact of how well the zero-flows are predicted rather than how well the models predict the exact sizes of the other flows. Fig. 4 addresses this by considering the deviance values for the models compared against truncated data sets, in which only flows above a certain $F_\text {min}$ are considered. The figure shows that the trends are almost completely as above. The only exception is for flows greater than 10,000 predicted by model A (‘Populations’). This model uses the largest weights and therefore overestimates most flows, but predicts more reasonable values for the larger flows. This suggests larger flows are therefore systematically underestimated by the other models. However, only $0.022\%$ of flows predicted by model A are greater than 10,000, so this trend does not significantly affect the validity of our overall conclusions.

**Figure 5:**
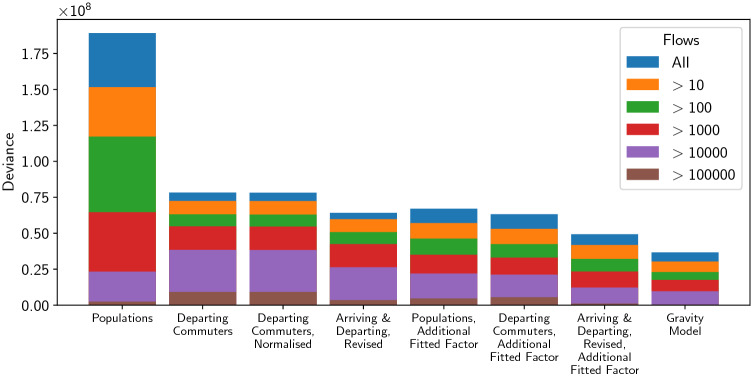
Deviance values Eq. (8) for radiation models A, B, C, E, F, G, H (from left to right) described in Table 1, alongside the production constrained gravity model of Eq. (2), with data sets that are truncated using the minimum values shown in the legend. Lower values represent better models. These data are from the American Commuter Survey 2009–2013^3^. For each model the top of a coloured bar represents the deviance value for that model when the data is limited to flows above the value indicated in the legend.

Finally, we consider the second data set (the American Commuter Survey^3^ 2009–2013). In Fig. 5 we show the deviance values for these data, though the trends are the same in all our measures. The results for this data reinforce all of our conclusions.

## Conclusions and discussion

For our data on modern US commuter flows, the most accurate flow predictions came from the production-constrained gravity model. Looking at the truly “parameter free” radiation models, that is models with no fitted global parameters, radiation model E (‘Arriving & Departing, Revised’) was most successful. The set of parameter free radiation models A–E showed that matching each model parameter to an appropriate data value improves the model performance as we should expect. This radiation model E benefits from a number of improvements over the original radiation model: choosing an appropriate input data set (e.g. the number of individuals who leave each site rather than population); correctly adjusting the model to include measures of both attractiveness and repulsiveness for each site; and introducing the correct normalisation.

Another conclusion was that adding an additional global parameter, and setting that parameter by finding the best fit, improves the performance of any model. The penalty of having an extra parameter is negligible for our data sets while there is vast room for improvement in what are poor fits in statistical terms. This is why the radiation model that best fits both data sets is radiation model H. This is the same as radiation model E, but with a single additional fitted parameter.

Despite these improvements, and in direct contrast with the results elsewhere^2^, our statistical measures show that for these US commuting data sets the radiation model is vastly inferior to an appropriately chosen gravity model for most realistic purposes, i.e. where there is data that can be used to fit parameters—what appears to be a small visual difference between models in our plots represents a large numerical difference.

The relative success of our chosen gravity model highlights another result. The use of a gravity model on the same data^2^ made less successful predictions than the radiation model in spite of its having nine fitted parameters to the latter’s zero. This underscores the importance of constraints, and the requirement that only models with corresponding constraints be compared against each other when the impact of these constraints is not the topic of investigation. This is why in this work all our models are production constrained in order to make our comparisons fair.

By examining the deviance values, we further established that none of these models fit our data well in an absolute sense. This is unsurprising: the large number of factors affecting commuter flows—geographical and socio-economic—limit the extent to which a simple model with very few parameters could make accurate predictions.

Our work leads us to make recommendations for spatial interaction modelling in general. First, we suggest that non-Gaussian regression (in particular Poisson regression) as applied to log-likelihood, Bayesian information criterion and deviance, are good statistical methods to use when analysing spatial interaction models. These have a firm theoretical grounding and provide an unbiased statistical approach. Second, we should ensure any feature that is not being explicitly tested is controlled for. Here, this means all our models enforce the production constraint. In fact, it would be trivial to add the input constraint into all these models, as is standard for gravity models^7^. Such an improvement requires no additional parameters. Third, the small penalty in the Bayesian information criterion arising from additional parameters, as well as the lower deviance values of models with fitted parameters, attest to the fact that if data exist that can be used for fitting, then a model with many physically relevant parameters can be improved by fitting to this data. Having such fitted model parameters is an advantage, not a disadvantage. Fourth, models should make use of as much available information as possible. We found that if we used the actual commuter flows in and out of sites in a way that matched that narrative behind a model, then results were better than trying to use the population as some proxy for the actual flows. Lastly, these simple spatial interaction models should be used only to provide an outline of real-world processes, with fitted parameter values giving general insights into spatially-constrained processes. These models are only ever crude approximations of reality.

## Supplementary information

Supplementary Information.

## Data Availability

All the data used in this work is publicly available as cited within the text^1,3–6^.
