# Supplementary material for: Predictive limitations of spatial interaction models: a non-Gaussian analysis
Source: Sci Rep. 2020 Oct 15;10:17474. doi: 10.1038/s41598-020-74601-z (PMC7566590; doi:10.1038/s41598-020-74601-z)
Supplement: Supplementary file 1 — Supplementary Information. [file 41598_2020_74601_MOESM1_ESM.pdf]

Supplementary Information for

Predictive Limitations of Spatial Interaction Models: A Non-Gaussian Analysis

B. Hilton*, A. P. Sood*, T. S. Evans,

(*) Equal first authors

Centre for Complexity Science, and Theoretical Physics Group,
Physics Dept., Imperial College London, SW7 2AZ, U.K.

4th September 2020

A Summary of Notation

A summary of the notation used in this work is given in Table A1.

Notation	Meaning
i, j	Indices of sites.
P_i	The population of site i.
O_i	The number of commuters leaving site i.
I_i	The number of commuters arriving at site i.
N_c	The total number of commuters in the data. This satisfies $N_c = \sum_i O_i = \sum_j I_j$.
F_{ij}	The actual flow from a source site i to a target site j as found in the data.
d_{ij}	A measure of the distance from site i to site j.
$\widehat{F}_{ij}$	The estimated flow from a source site i to a target site j as predicted by some model.
w_i	The site 'weight' model parameter. Controls the flow into and out of a site.
m_i	The site 'aspiration' model parameter. Controls the distribution of flows from site i.
n_i	The site 'attractiveness' model parameter, the number of 'opportunities' . Controls flow into site i.
t_i	Model parameter controlling the total flow leaving site i (site 'repulsiveness').

Table A1: A summary of the different data values (top half) and the different model parameters (bottom half) used in this paper.

B Versions of the Radiation model

In this section we give explicit forms for the Radiation models used in our work written in terms of the actual data values used. These are summarised in Table B2 (reproduced from the main text) with detailed equation given in the following subsections. In each case we explain how the parameters m_i, n_j and t_i of the Radiation model are replaced by values obtained with data. For simplicity, we will repeat here our standard form for the Radiation model:-

$$\widehat{F}_{ij} = t_i \frac{m_i n_j}{(m_i + s_{ij})(m_i + n_j + s_{ij})} . \tag{B.1}$$

We will also note any normalisation factors used [1], something not included in the simple form (B.1) above. For completeness this is

$$\widehat{F}_{ij} = \left(\frac{N_c}{N_c - m_i} \right) t_i \frac{m_i n_j}{(m_i + s_{ij})(m_i + n_j + s_{ij})} . \tag{B.2}$$

Here $N_c = \sum_i n_i$ is the total number of opportunities in the system.

	Name	$\mathbf{m_i}$	$\mathbf{n_i}$	$\mathbf{t_i}$	Normalised?	Eq.
A.	Total population	P_i	P_i	P_i	×	(B.3)
B.	Departing commuters	O_i	O_i	O_i	×	(B.4)
C.	Departing commuters, Normalised	O_i	O_i	O_i	✓	(B.5)
D.	Arriving & Departing, Naïve split	O_i	I_i	O_i	×	(B.6)
E.	Arriving & Departing, Revised split	I_i	I_i	O_i	✓	(B.7)
F.	Total population, Fitted factor	P_i	P_i	αP_i	×	(B.8)
G.	Departing commuters, Fitted factor	O_i	O_i	αO_i	×	(B.9)
H.	Arriving & Departing, Revised, Fit factor	I_i	I_i	αO_i	✓	(B.10)

Table B2: A summary of the different versions of the radiation model. The 'Normalised?' column indicates if a model uses a normalisation that enforces the production constraint exactly (B.2), a cross indicates that (B.1) is used for that model. In each case we specify which of the site data values, (P_i population, I_i commuters arriving, O_i commuters leaving) is used for the model site parameters (aspirations m_i, opportunities n_i, out flow t_i). See Table A1 for a summary of the notation.

B.1 The Populations Radiation model

The 'Populations' model (model A) is a standard radiation model (B.1) that sets all input parameters equal to the population ($m_i = n_i = t_i = P_i$). This gives us that

$$\widehat{F}_{ij} = P_i \frac{P_i P_j}{(P_i + s_{ij})(P_i + s_{ij} + P_j)} . \tag{B.3}$$

Here the intervening opportunities measure s_{ij} is the total population of sites lying closer to site i than site j (excluding site i itself).

B.2 The Departing Commuters Radiation model

The 'Departing Commuters' model (model B) is a standard radiation model (B.1) defined as

$$\widehat{F}_{ij} = O_i \frac{O_i O_j}{(O_i + s_{ij})(O_i + s_{ij} + O_j)} , \tag{B.4}$$

Here all input parameters to the number of commuters who depart from site i, $m_i = n_i = t_i = O_i$. The intervening opportunities measure s_{ij} in this model is the total number of commuters leaving all sites that are closer to site i than site j (excluding the commuters leaving site i itself). Note that in this model the total flow leaving site i is *not* equal to the number of commuters leaving site i, $\sum_{j \in \mathcal{T}_i} \widehat{F}_{ij} \neq O_i$. This model has failed this normalisation criteria but in many cases this can be a small effect so this is not an unreasonable model to use.

B.3 The Normalised Departing Commuters Radiation model

The 'Departing Commuters, Normalised' model (model C) is a normalised Radiation model (B.2) defined as

$$\widehat{F}_{ij} = \left(\frac{O_i}{1 - O_i/N_c} \right) \frac{O_i O_j}{(O_i + s_{ij})(O_i + s_{ij} + O_j)} , \qquad N_c = \sum_i O_i \tag{B.5}$$

Again all input parameters to the number of commuters who depart from site i, $m_i = n_i = t_i = O_i$. The intervening opportunities measure s_{ij} is given in terms of the outputs of intervening sites, exactly as in the Departing Commuters Radiation model (model B) (B.4). Unlike that model, this version is normalised properly so the total flow out of the model equals the associated data value exactly, $\sum_{j \in \mathcal{T}_i} \widehat{F}_{ij} = O_i$ (see (B.2)).

B.4 The Arriving & Departing, Naïve Split, Radiation Model

The 'Arriving & Departing, Naïve Split' radiation model (model D) is based on (B.1), and defined as

$$\widehat{F}_{ij} = O_i \frac{O_i I_j}{(O_i + s_{ij})(O_i + s_{ij} + I_j)} . \tag{B.6}$$

Here both m_i and t_i (see (B.1)) are set equal to the number of commuters who depart from site i so $m_i = t_i = O_i$. We set the attractiveness model parameter, the number of opportunities at site j, to be equal to the total number of commuters found in the data to be arriving at site j, so $n_j = I_j$. This last identification then means that the intervening opportunities measure s_{ij} has to be the cumulative number of commuters arriving at all sites closer to i than j (excluding site i), regardless of their origin.

B.5 The Arriving & Departing, Revised, Radiation Model

The 'Arriving & Departing, Revised' model (model E) is a normalised Radiation model (B.2) defined as

$$\widehat{F}_{ij} = \left(\frac{N_c}{N_c - I_i}\right) O_i \frac{I_i I_j}{(I_i + s_{ij})(I_i + s_{ij} + I_j)}, \qquad N_c = \sum_i I_i . \tag{B.7}$$

Here at each site i the site attractiveness parameter n_i and site aspiration parameter m_i are both set equal to the number of commuters arriving at site i, $m_i = n_i = I_i$. The site repulsiveness parameter t_i is set equal to the number of commuters leaving a site O_i and the normalisation factor here ensures this is equal to the total flow predicted from the model, $\sum_j \widehat{F}_{ij} = O_i$. The intervening opportunities measure s_{ij} in this model is the total number of commuters arriving (I_i) at all sites that are closer to site i than site j (excluding the commuters arriving at site i itself).

B.6 The Populations, Additional Fitted Factor, Radiation model

The 'Populations, Additional Fitted Factor' model (model F) is a standard radiation model (B.1) defined as

$$\widehat{F}_{ij} = \alpha P_i \frac{P_i P_j}{(P_i + s_{ij})(P_i + s_{ij} + P_j)} . \tag{B.8}$$

Here we have $m_i = n_i = P_i$, but the flow parameter t_i is set proportional to the total population $t_i = \alpha P_i$. This α is a single additional parameter found by optimising the fit to the data using a maximum likelihood estimation. The intervening opportunities measure s_{ij} is the total population of sites lying closer to site i than site j (excluding site i itself).

B.7 The Departing Commuters, Additional Fitted Factor, Radiation model

The 'Departing Commuters, Additional Fitted Factor' model (model G) is a standard radiation model (B.1) defined as

$$\widehat{F}_{ij} = \alpha O_i \frac{O_i O_j}{(O_i + s_{ij})(O_i + s_{ij} + O_j)} . \tag{B.9}$$

Here we have set $m_i = n_i = O_i$, the number of commuters who depart from site i, and $t_i = \alpha O_i$ where α is a single fitted parameter. The intervening opportunities measure s_{ij} is therefore the sum of all the outputs, O_i, of intervening sites. Note that this model is not normalised, so $\sum_j \widehat{F}_{ij} \neq \alpha O_i$ and so in turn $\alpha = 1$ is not to be expected even in 'perfect' data generated from the model itself. In reality, the lack of accuracy in the model predictions is likely to ensure some $\alpha \neq 1$ will provide an optimal fit to the data.

B.8 The Arriving & Departing, Revised, Additional Fitted Factor, Radiation model

The 'Arriving & Departing, Revised, Additional Fitted Factor' model (model H) is a normalised Radiation model (B.2) defined as

$$\widehat{F}_{ij} = \alpha O_i \left(\frac{N_c}{N_c - I_i}\right) \frac{I_i I_j}{(I_i + s_{ij})(I_i + s_{ij} + I_j)}, \qquad N_c = \sum_i I_i \tag{B.10}$$

Here we have set $m_i = n_i = I_i$ and $t_i = \alpha O_i$. This model is a normalised radiation (B.2). The intervening opportunities measure s_{ij} is the total number of commuters arriving at all sites closer to i than j.

The model is normalised so in principle we might expect $\alpha = 1$. However we leave α as a single parameter to be found by optimising the fit to the data and so $\alpha \neq 1$ is likely given the inevitable imperfections in this simple model.

C Gravity Models

The Gravity model used Simini et al. [2] was

$$\widehat{F}_{ij} = \theta(D - d_{ij}) C_1 (P_i)^{\alpha_1} (P_j)^{\beta_1} (d_{ij})^{-\gamma_1} + \theta(d_{ij} - D) C_2 (P_i)^{\alpha_2} (P_j)^{\beta_2} (d_{ij})^{-\gamma_2} , \qquad \text{(C.1)}$$

and similar with the exponential form for the deterrence function. The Heaviside theta functions split the model into short range and long range forms with a global model parameter D determining the distance scale. This is a model with nine global parameters found through finding a best fit to the data. We have not used this form in our work. We have instead worked with a production constrained gravity model with one parameter (B.2) as this matches the approximate production constraint and the single parameter used in the Radiation model of Simini et al. [2] (the same as our Radiation model F of (B.8), see Table B2).

D Applying common statistical techniques for comparing models

In this section, we discuss some of the commonly used statistical techniques for comparing models further, and apply these techniques to compare the models listed in Appendix B and the production constrained gravity model described by

$$\widehat{F}_{ij} = \frac{t_i n_j d_{ij}^{-\beta}}{\sum_k n_k d_{ik}^{-\beta}} . \quad (i \neq j) \qquad \text{(D.1)}$$

It should be noted that any fitted parameters associated with the models have been calculated, as in the main text, through maximum likelihood estimation that utilises Poisson regression: only the techniques used for model comparison are the standard methods from the literature.

It should be noted that for most statistical methods, models whose predictions perfectly match real data can be identified even if the techniques are not technically theoretically justifiable. For example, a perfect model will always have DSC $= R^2 = 1$. As such, we should expect some correlation between the results from these techniques and the results using our suggested, more accurate, methodology. However, in some cases, these techniques will give results that can be difficult to interpret – or worse, will give wrong answers with no indication that the error is occurring.

D.1 Sørensen-Dice coefficient

The Sørensen-Dice coefficient can be applied to flows in the context of spatial modelling where it is sometimes referred to as the 'common part of commuters' [3, 4]. It measures the overlap between the predicted and actual flow between each pair of sites, and is given by

$$\mathrm{DSC} = \frac{\sum_{ij} \min(\widehat{F}_{ij}, F_{ij})}{\sum_{ij} F_{ij}}. \tag{D.2}$$

It thus gives a result between 0 and 1, with a Sørensen-Dice coefficient of 1 representing a model with perfect predictions and 0 a model with very weak predictions. The Sørensen-Dice coefficient has been applied to spatial data in many cases, for instance [3, 4, 5, 6, 7, 8, 9, 10, 11].

One of the key limitations of the Sørensen-Dice coefficient is that, in this form, it does not apply when the total predicted flow is not fixed to the total real flow (e.g. by a production constraint or weaker 'total-flow' constraint). This is because without such a constraint, a model could predict infinite flows between all pairs of sites and obtain a perfect Sørensen-Dice coefficient (equal to 1). Perhaps most significantly, small percentage deviations in the predictions of large flows will have a more significant impact on the Sørensen-Dice coefficient than large percentage deviations of small flows; the Sørensen-Dice coefficient could more strongly penalise a model that consistently predicts flows to within 10% of their actual value than a model which predicts its largest flows accurately but overestimates tiny flows by several orders of magnitude. Thus, models which make better overall predictions, when judged by a more rigorous Poisson regression analysis, can have a worse Sørensen-Dice coefficient, and, additionally, differences between the Sørensen-Dice coefficients of different models can be difficult to interpret.

Variations on the form of the DSC can resolve the first issue, by multiplying the numerator by two and adding $\sum_{ij} \widehat{F}_{ij}$ to the denominator [1, 12], perhaps applied only to links with non-zero values, the 'common part of links' measure used in [7]. However, this amendment does not resolve the other limitations.

Sørensen-Dice coefficients for the models we have analysed appear in Fig. D1. The results would indicate that model A ('Populations') performs best by this measure, however since this model lacks a total flow constraint, the associated Sørensen-Dice coefficient is not meaningful. While some of the Sørensen-Dice coefficient results follow the same pattern as those found from our maximum likelihood methods, with model B ('Departing Commuters') being the weakest of the models shown (excluding model A), and the gravity model giving the best result, some of the results are dissimilar. For example, by our methods model E is shown to have greater predictive power than model F, which is not captured by the Sørensen-Dice coefficient.

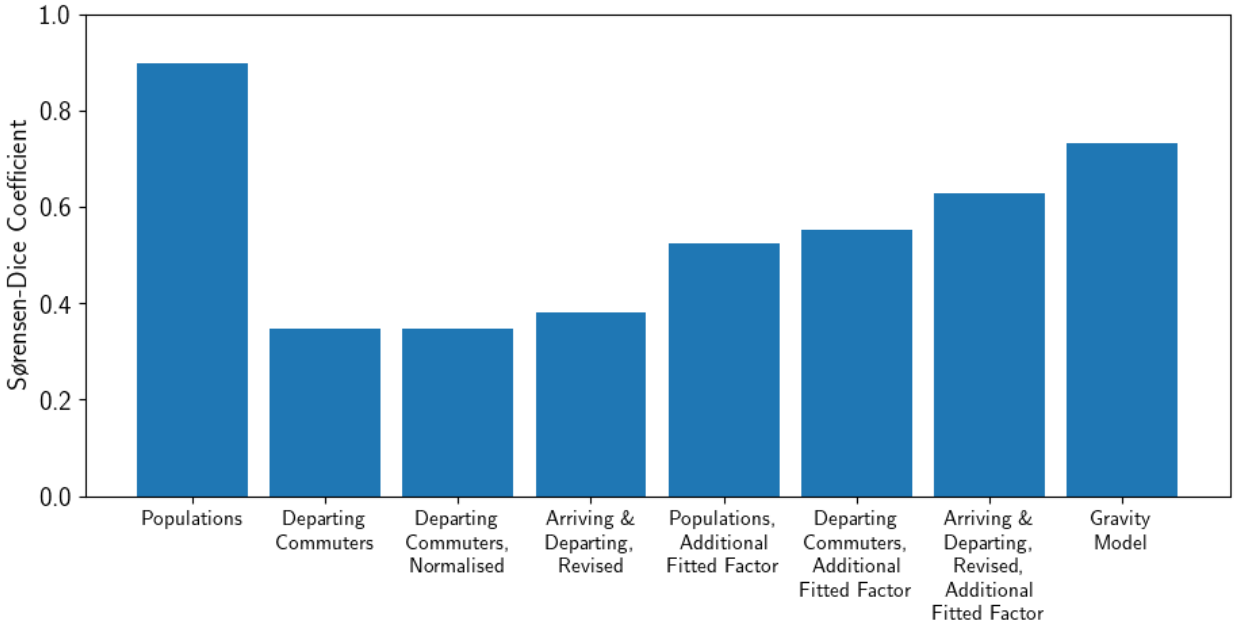

Figure D1: Sørensen-Dice coefficient values (D.2) for radiation models A, B, C, E, F, G, H (from left to right) described in Table B2, alongside the production constrained gravity model of (D.1). Higher values represent better models. These data are from the US Census 2000 [13].

D.2 Kolmogorov-Smirnov Test

The Kolmogorov-Smirnov test is defined [14]:

$$K = \sup |\widehat{F}_{ij} - F_{ij}|. \tag{D.3}$$

This is a nonparametric test of the equality of two continuous valued functions, which in our case are the data and the model prediction of the flow between each pair of sites. The test may return values between 0 and $+\infty$, with smaller values representing better models. While for pairs of sites with high flows, the integer value may be reasonably approximated by a continuous function, the integer valued nature of the data will be a concern for the majority of site pairs where flows are low.

However our main concern is that the Kolmogorov-Smirnov test requires its two input functions to be independent. When the Kolmogorov-Smirnov test is used in spatial modelling [15], the test is usually applied to a model whose parameters have been estimated by fitting to the same data so not the data and model functions are not independent [14]. When the conditions for the validity of this test are not met, the results can appear absurd. For example, when comparing two models, one of which is very accurate, and one of which is not, but both of which have a single large outlier, the Kolmogorov-Smirnov test will return large values for both models.

The results found by applying the Kolmogorov-Smirnov test to our models are shown in Fig. D2. As with the Sørensen-Dice coefficient, there is some correlation between the ranking of models obtained using the Kolmogorov-Smirnov statistic and the ranking of models obtained using our more rigourous analysis above. For example, model A ('Populations') is shown to be the worst of the models depicted in this figure. However, there are also notable differences – the gravity model is, according to the Kolmogorov-Smirnov test, worse than model H ('Arriving & Departing, Revised, Additional Fitted Factor).

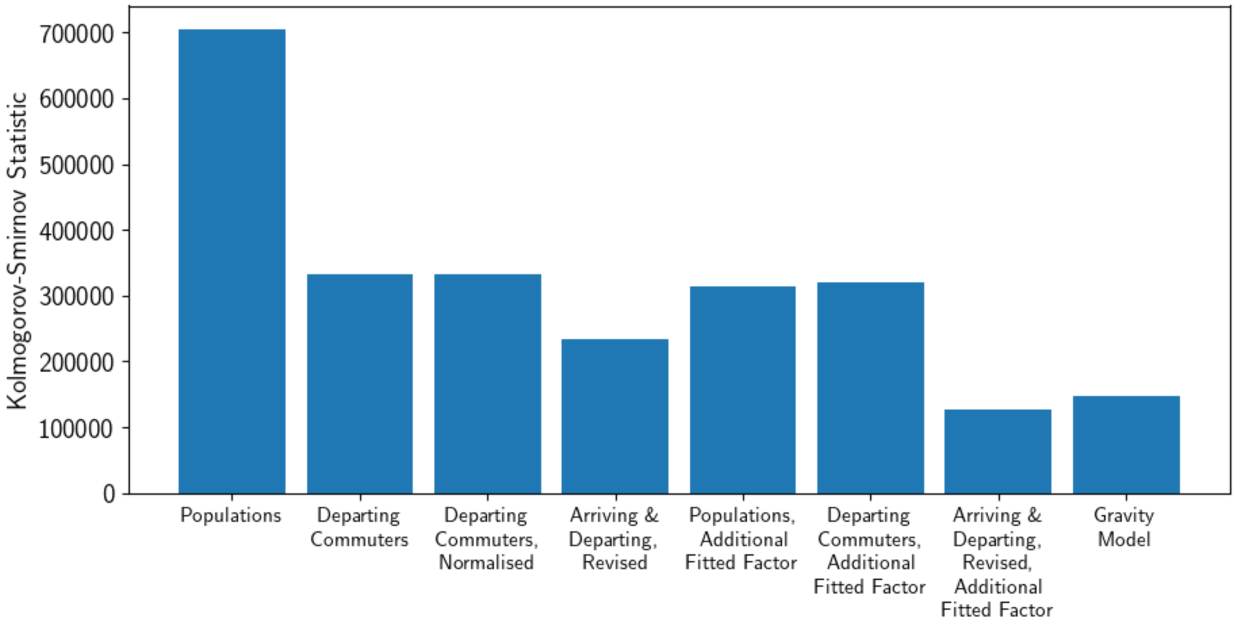

Figure D2: Kolmogorov-Smirnov values (D.3) for radiation models A, B, C, E, F, G, H (from left to right) described in Table B2, alongside the production constrained gravity model of (D.1). Lower values represent better models. These data are from the US Census 2000 [13].

D.3 The Coefficient of Determination, R^2

The coefficient of determination R^2 is one of a family of measures which assume that the error distribution $p(F_{ij}|\widehat{F_{ij}})$ is Gaussian for any i, j. However, it is very common that real data sets feature no negative flows and very many small flows, meaning that the central limit theorem does not apply and the distributions of flows between any pair of sites cannot be assumed to be Gaussian. As such R^2 should not be assumed to be a theoretically valid measure when used to analyse predictions from spatial interaction models.

The coefficient of determination is given by

$$R^2 = 1 - \frac{\sum_{ij}(F_{ij} - \hat{F}_{ij})^2}{\sum_{ij}(F_{ij} - \bar{F})^2}, \tag{D.4}$$

where $\bar{F}$ is the mean value of the data set. R^2 values can range from $-\infty$ to 1, with values closer to 1 representing better models. The R^2 values obtained for our models are shown in Fig. D3. This measure has been used to assess the goodness-of-fit of models to data in spatial contexts [1, 16, 17].

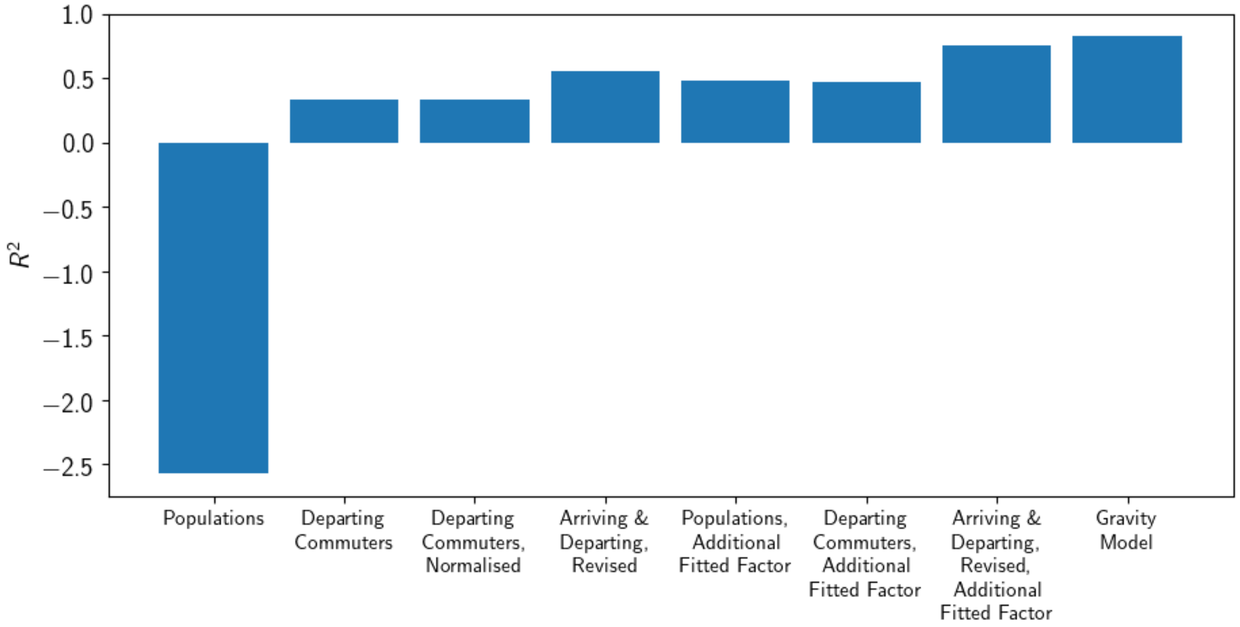

Figure D3: R^2 values (D.4) for radiation models A, B, C, E, F, G, H (from left to right) described in Table B2, alongside the production constrained gravity model of (D.1). Lower values represent better models. These data are from the US Census 2000 [13].

The first thing to note is that whilst, in general, models shown to be better in our more rigourous analysis above are better using R^2 values, there are key differences in the rankings. For example, model F ('Populations, Additional Fitted Factor') and model G ('Departing Commuters, Additional Fitted Factor') are, according to these R^2 values, more successful than model E ('Arriving & Departing, Revised').

Most notably, model A ('Populations') has a negative R^2. Usually, this would mean that the model being tested is worse than the null hypothesis — for R^2, the null hypothesis is a horizontal line. However, in the (theoretically unjustifiable) way in which the R^2 has been used here, we cannot easily interpret the negative R^2 in this way. This is illustrative of the general problem of using statistical methods that are not theoretically justifiable in the case of spatial interaction modelling — it is very difficult to legitimately interpret the values obtained.

There are other similar measures based on squared differences, so typically motivated by Gaussian statistics, and these include mean squared errors [18] and Pearson correlation coefficients [19, 20]. We do not pursue them here.

E Data

The distribution of commuter flows in the US Census 2000 [13] data is shown in Table E3.

Flow	Number
All	9665881
> 0	164764
> 10	77432
> 100	21237
$> 1,000$	7058
$> 10,000$	1814
$> 100,000$	212

Table E3: The number of county-county pairs with flows equal to or greater than the flow minimum given. Data for county-county commuter numbers is as given in the US Census 2000 [13].

References

[1] Masucci, A. P., Serras, J., Johansson, A. & Batty, M.
Gravity versus radiation models: On the importance of scale and heterogeneity in commuting flows.
Physical Review E **88**, 022812, DOI: 10.1103/PhysRevE.88.022812 (2013).

[2] Simini, F., Gonzalez, M. C., Maritan, A. & Barabasi, A.-L.
A universal model for mobility and migration patterns.
Nature **484**, 96–100, DOI: 10.1038/nature10856 (2012).

[3] Gargiulo, F., Lenormand, M., Huet, S. & Baqueiro Espinosa, O.
Commuting network models: Getting the essentials.
Journal of Artificial Societies and Social Simulation **15**, 6– (2012).

[4] Lenormand, M., Huet, S., Gargiulo, F. & Deffuant, G.
A universal model of commuting networks.
PLOS ONE **7**, e45985, DOI: 10.1371/journal.pone.0045985 (2012).
1203.5184v2.

[5] Wesolowski, A., O'Meara, W. P., Eagle, N., Tatem, A. J. & Buckee, C. O.
Evaluating spatial interaction models for regional mobility in sub-saharan africa.
PLOS Computational Biology **11**, e1004267, DOI: 10.1371/journal.pcbi.1004267 (2015).

[6] Kang, C., Liu, Y., Guo, D. & Qin, K.
A generalized radiation model for human mobility: Spatial scale, searching direction and trip constraint.
PLOS ONE **10**, e0143500, DOI: 10.1371/journal.pone.0143500 (2015).

[7] Lenormand, M., Bassolas, A. & Ramasco, J. J.
Systematic comparison of trip distribution laws and models.
Journal of Transport Geography **51**, 158–169, DOI: 10.1016/j.jtrangeo.2015.12.008 (2016).

[8] Grauwin, S. *et al.*
Identifying and modeling the structural discontinuities of human interactions.
Scientific Reports **7**, DOI: 10.1038/srep46677 (2017).

[9] Yan, X.-Y. & Zhou, T.
Destination choice game: A spatial interaction theory on human mobility.
Scientific Reports **9**, DOI: 10.1038/s41598-019-46026-w (2019).

[10] Liu, E.-J. & Yan, X.-Y.
A universal opportunity model for human mobility.
Scientific Reports **10**, 4657, DOI: 10.1038/s41598-020-61613-y (2020).

[11] Yao, X. *et al.*
Origin-destination flow, data imputation, spatial interaction network, graph embedding, graph convolution; spatial origin-destination flow imputation using graph convolutional networks.
IEEE Transactions on Intelligent Transportation Systems 1–11, DOI: 10.1109/tits.2020.3003310 (2020).

[12] Yang, Y., Herrera, C., Eagle, N. & González, M. C.
Limits of predictability in commuting flows in the absence of data for calibration.
Scientific Reports **4**, 5662, DOI: 10.1038/srep05662 (2014).

[13] United States Census Bureau.
County-to-county worker flow files (2001).

[14] Steinskog, D. J., Tjøstheim, D. B. & Kvamstø, N. G.
A cautionary note on the use of the Kolmogorov–Smirnov test for normality.
Monthly Weather Review **135**, 1151–1157 (2007).

[15] Kang, C., Liu, Y., Guo, D. & Qin, K.
A Generalized Radiation Model for Human Mobility: Spatial Scale, Searching Direction and Trip Constraint.
PLOS One **10** (2015).

[16] Hong, I. & Jung, W.-S.
Application of gravity model on the korean urban bus network.
Physica A: Statistical Mechanics and its Applications **462**, 48–55, DOI: 10.1016/j.physa.2016.06.055 (2016).

[17] Beiró, M. G., Panisson, A., Tizzoni, M. & Cattuto, C.
Predicting human mobility through the assimilation of social media traces into mobility models.
EPJ Data Science **5**, DOI: 10.1140/epjds/s13688-016-0092-2 (2016).

[18] Curiel, R. P., Pappalardo, L., Gabrielli, L. & Bishop, S. R.
Gravity and scaling laws of city to city migration.
PLOS ONE **13**, e0199892, DOI: 10.1371/journal.pone.0199892 (2018).

[19] Liu, J., Zhao, K., Khan, S., Cameron, M. & Jurdak, R.
Multi-scale population and mobility estimation with geo-tagged tweets.
In *2015 31st IEEE International Conference on Data Engineering Workshops*, DOI: 10.1109/icdew.2015.7129551 (IEEE, 2015).

[20] Giles, J. R. *et al.*
The duration of travel impacts the spatial dynamics of infectious diseases.
Proceedings of the National Academy of Sciences 201922663, DOI: 10.1073/pnas.1922663117 (2020).
